# Complete Genome Sequences of *Helicoverpa armigera* Single Nucleopolyhedrovirus Strains AC53 and H25EA1 from Australia

**DOI:** 10.1128/genomeA.01083-15

**Published:** 2015-09-24

**Authors:** Christopher Noune, Caroline Hauxwell

**Affiliations:** School of Earth, Environmental and Biological Sciences, Science and Engineering Faculty, Queensland University of Technology, Brisbane, Australia

## Abstract

We report here the genome sequences of two alphabaculoviruses of *Helicoverpa* spp. from Australia: AC53, used in the biopesticides ViVUS and ViVUS Max, and H25EA1, used in *in vitro* production studies.

## GENOME ANNOUNCEMENT

*Helicoverpa* spp. (Lepidoptera, Noctuidae) are polyphagous pests of international significance (1). Widespread resistance to chemical insecticides has prompted the registration of biopesticides based on baculoviruses (*Baculoviridae*) (2).

Two species of group II nucleopolyhedroviruses (genus *Alphabaculovirus*) from *Helicoverpa* species have been designated *Helicoverpa armigera* single nucleopolyhedrovirus (HaSNPV) and *Helicoverpa zea* single nucleopolyhedrovirus (HzSNPV) (3–11). Strain AC53 (also known as A44WT [11–13]) is used in the biopesticides ViVUS and ViVUS Max (AgBiTech Pty. Ltd.) (2). It was originally isolated from an unspecified *Helicoverpa* species from a cadaver from Brookstead, Southeast Queensland, Australia, in 1974 (2, 11–13) and isolate P9/H25WT from an unspecified *Helicoverpa* species from a cadaver from Central Queensland in 1973 (14–19). Both isolates were passaged initially through *Helicoverpa punctigera* Wallengren and then repeatedly through *H. armigera* (Hübner) by the Queensland Department of Primary Industries (DAFF Qld); strain H25EA1, used *in vitro* baculovirus production, was selected *in vitro* by CSIRO from P9/H25WT (14–19).

AC53 (AgBiTech Pty. Ltd.) and H25EA1 (from S. Reid, University of Queensland) were passaged once through *H. armigera* larvae. Viral DNA was extracted from occlusion bodies, as previously described (7, 20), and sequenced using the Ion Torrent PGM (316 Chip, 200-bp chemistry). Read quality was determined using FastQC 0.11.2 (http://www.bioinformatics.babraham.ac.uk/projects/fastqc/) and the qProfiler tool from the AdamaJava project (Queensland Centre for Medical Genomics) and trimmed using CLC Genomics Workbench 7.04 (CLC 7.04), with a final Phred score of 28.

AC53 contigs were assembled *de novo* using CLC 7.04 and compared with BLAST against all available *Helicoverpa* species SNPV genomes (GenBank accession numbers JN584482, NC011354, NC003349, NC003094, and NC002645). The HzSNPV (accession no. NC003349) genome was selected as a mapping reference and a consensus sequence for AC53 produced using Burrows-Wheeler aligner (BWA)-mem 0.7.5a, SAMtools 0.19, and the genome analysis toolkit GATK 3.1-1. *De novo* contigs were assembled to fill gaps (21–25). Assembly of the H25EA1 genome was conducted according to the same process for mapping to the AC53 sequence.

The AC53 and H25EA1 genomes were, 130,442 bp and 130,440 bp, with G+C contents of 39.2% and 39.1%, respectively. The homology between the strains was 99.60%. The homology to HzSNPV (accession no. NC003349) was 99.56% but ranged between 98.43% (accession no. NC003094) and 98.99% (accession no. NC011354) in comparison to HaSNPV genomes.

Both strains contain 138 open reading frames (ORFs), 5 homologous repeat (Hr) regions, and all 62 of the conserved genes were found in all lepidopteran baculoviruses (26). Of the 138 ORFs, 52 had 100% sequence homology between the two strains. The greatest differences between AC53 and H25EA1 were found in the baculovirus repeated open reading frames BRO-A (89.78% homology), and BRO-B (96.41% homology) and the 5 Hr regions (94.61% to 99.27%). However, they contained 100% homology in the BRO region located at ORF107. This is consistent with many baculoviruses (27–29).

Both isolates contained the HaSNPV ORF42 (typically located at ORF43 in HzSNPVs) (30, 31), but unlike published HaSNPV genomes, both contained the HzSNPV ORF79 (7, 28), located at ORF78.

We conclude that AC53 and H25EA1 are type II *Heliothine* SNPVs intermediate between HzSNPV and HaSNPV and support the argument that all *Heliothine* SNPVs are variants of a single species of HaSNPV (3–5, 7).

### Nucleotide sequence accession numbers.

The complete sequences of HaSNPV AC53 and HaSNPV H25EA1 were deposited to GenBank under the accession numbers KJ909666 and KJ922128, respectively.
